# Supplementary material for: Metabolomics of Cerebrospinal Fluid Amino and Fatty Acids in Early Stages of Multiple Sclerosis
Source: Int J Mol Sci. 2023 Nov 13;24(22):16271. doi: 10.3390/ijms242216271 (PMC10671192; doi:10.3390/ijms242216271)
Supplement: Supplementary file 1 [file ijms-24-16271-s001.zip › ijms-2569343-supplementary.pdf]

Supplementary Materials

Supp. Table S1: Basic CSF cytological and biochemical results of the MS group

ID	Cerebrospinal fluid								Time from clinical symptoms to CSF collection (months)
	IEF IgG	IEF IgA	IEF IgM	IEF Kappa	IEF Lambda	Protein (g/l)	Glc (mmol/l)	Mononucl (µl)	
**1**	5	3	4	2	1	0.31	5.5	19	0.3
**2**	7	10	9	8	5	0.32	3.85	4	0.7
**3**	7	11	21	10	2	0.26	3.31	40	0.2
**4**	9	9	9	8	7	0.25	3.12	12	1
**5**	1	1	1	0	0	0.27	3.10	2	1.5
**6**	13	11	9	15	12	0.26	3.44	30	1
**7**	17	11	22	16	11	0.32	3.41	4	N/A
**8**	3	10	8	10	8	0.15	3.35	8	3
**9**	2	2	0	6	1	0.43	3.31	27	1
**10**	14	12	5	12	0	0.25	3.36	2	60
**11**	9	8	8	7	8	0.41	3.04	13	0.5
**12**	6	8	8	8	0	0.43	3.11	34	6
**13**	10	7	5	9	10	0.45	2.94	42	1.5
**14**	10	9	4	4	8	0.19	3.85	2	0.1
**15**	16	14	13	16	6	0.40	3.72	22	0.7
**16**	12	4	7	2	0	0.20	3.27	30	0.7
**17**	15	0	8	6	0	0.33	2.75	5	1
**18**	9	14	14	11	10	0.37	3.21	2	3
**19**	2	1	3	3	6	0.21	3.47	3	24
**20**	8	9	6	3	12	0.46	3.31	20	4
**21**	5	9	6	5	4	0.18	3.03	1	24
**22**	9	14	10	2	2	0.23	3.92	22	0.7
**23**	0	0	0	3	1	0.15	3.69	1	0.5
**24**	4	3	2	2	3	0.21	3.36	3	0.3
**25**	0	0	0	0	2	0.25	3.32	2	48
**26**	2	0	0	4	4	0.46	5.62	8	4
**27**	5	2	8	12	4	0.11	2.96	4	2
**28**	7	4	3	3	1	0.15	3.62	10	2
**29**	7	9	7	5	3	0.4	3.58	13	N/A
**30**	5	5	1	6	7	0.36	3.79	6	1
**31**	2	0	0	0	0	0.31	3.04	3	0.3
**32**	0	0	1	1	1	0.37	3.24	5	1
**33**	6	0	1	0	0	0.32	3.1	2	3
**34**	18	12	9	10	6	0.27	3.27	3	4
**35**	7	3	0	1	0	0.27	3.31	2	3.3
**36**	10	10	7	9	6	0.49	3.85	13	0.3
**37**	3	0	1	0	3	0.22	3.26	6	1.7
**38**	15	16	0	1	9	0.38	3.01	12	0.7
**39**	19	15	10	1	13	0.28	3.29	20	1.5
**40**	13	0	4	2	13	0.16	3.12	4	0.2

Note: ID = identification number; IEF = isoelectric focusing; Glc = glucose; Mononucl = number of mononuclear cells; N/A = non-available

Supp. Table S2: Clinical information of MS patients after the first clinical symptoms

ID	Age	Sex	Date of CSF collection	Medical history	Pharmacological history	EDSS			
						0	½ year	1 year	2 years

**1**	38	M	27.02.2017	-	-	1.5	1.5	1	1.5
**2**	26	F	28.03.2017	-	-	1	1	1	1
**3**	36	F	26.06.2018	Atopic eczema	-	2.5	3	2.5	2
**4**	27	F	04.10.2018	-	-	2.5	1.5	1	2
**5**	43	F	17.10.2018	Appendectomy Cholecystectomy	Hormonal anticonception	2	1.5	1.5	1.5
**6**	32	F	09.01.2019	Hypothyreosis	Levothyroxinum 50 µg daily	2.5	2	2	2
**7**	28	M	23.01.2019	-	-	1	N/A	N/A	N/A
**8**	27	F	13.03.2019	WPW syndrome	-	1	1.5	1.5	2
**9**	33	F	02.07.2019	NS sight impairment	-	1.5	N/A	N/A	N/A
**10**	40	F	13.11.2019	Hyperthyreosis	Levothyroxinum 50 µg daily	1.5	1.5	1.5	1.5
**11**	31	F	04.12.2019	-	Hormonal Anticonception	1.5	1.5	1.5	1.5
**12**	54	F	04.12.2019	Arterial hypertension, obesity, hypothyreosis	Levothyroxinum 137 mg, omeprazolum 20 mg, hydrochlorothiazidum 25 mg, allopurinolum 300 mg daily	2	2.5	2.5	2.5
**13**	18	F	30.12.2019	Asthma bronchiale, depression*	Hormonal Anticonception	2.5	2	2	2.5
**14**	25	F	27.02.2020	-	Hormonal anticonception	1.5	2	1.5	2
**15**	43	M	10.02.2020	-	-	1	N/A	N/A	N/A
**16**	25	F	29.04.2020	-	-	2.0	2	2	2
**17**	33	F	06.05.2020	-	-	1.5	N/A	N/A	N/A
**18**	40	F	24.08.2020	-	-	2	N/A	N/A	N/A
**19**	49	F	09.02.2021	Appendectomy	-	2.5	2.5	2.5	3
**20**	33	F	30.03.2021	-	-	1	1.5	1.5	1.5
**21**	43	F	06.04.2021	Gastroesophageal reflux	Hormonal anticonception, omeprazolum 20 mg daily	3.5	5.5	5.5	N/A
**22**	27	M	06.04.2021	-	-	1.5	2.0	2.0	2
**23**	28	F	13.04.2021	-	-	3.5	2.5	2	2
**24**	36	F	04.05.2021	-	-	2	2	2	2
**25**	27	M	05.05.2021	-	-	2.5	2.5	2.5	2.5
**26**	50	M	14.07.2021	-	-	1.5	1.5	1.5	1.5
**27**	26	F	05.10.2021	Hypothyreosis	Levothyroxinum 50 µg daily	1	1	1	1
**28**	29	F	11.10.2021	-	-	2	2	2	N/A
**29**	21	F	09.11.2021	-	-	0.5	0.5	0.5	N/A
**30**	41	F	01.12.2021	Gastroesophageal reflux	Omeprazolum 20 mg daily	1	N/A	N/A	N/A
**31**	39	F	12.06.2022	Leidens mutation, heterozygot	-	2	2	2	N/A
**32**	30	M	18.06.2022	-	-	1	N/A	N/A	N/A
**33**	42	F	16.08.2022	-	-	2	2	2	N/A
**34**	39	F	10.10.2022	-	Hormonal anticonception	1.5	1.5	1.5	N/A
**35**	49	M	25.10.2023	Radiotherapy of seminoma	-	3.5	3.5	3.5	N/A
**36**	39	M	03.11.2022	Arterial hypertension	Telmisartanum 40 mg, amlodipini besilas 10 mg, bisoprololi fumaras 10 mg daily	2	2	N/A	N/A
**37**	23	F	15.11.2022	-	-	0.5	0.5	N/A	N/A
**38**	29	F	13.12.2021	Hypothyreosis	Levothyroxinum 132 µg daily	3.5	4.0	4.0	N/A
**39**	19	F	21.12.2021	Asthma bronchiale	Salbutamoli sulfas 5 mg/ml when needed	1.5	1.5	2	N/A
**40**	24	F	12.01.2022	Extirpation of benign fibroma of	-	1	1	1.5	N/A

the left mamma

Note: ID = identification number; M = male; F = female; WPW = Wolf-Parkinson-White; NS = non-specific; N/A = non-available; * depression in this patient was without specific medication

Supp. Table S3: Descriptive statistics of metabolomic results with their counted concentrations

Metabolites	Arginine	Histidine	Choline	Serine	Tyrosine	Spermidine	Glutamate	Oleic acid	Stearic acid	Linoleic acid
**Mean MS**	3.9709	7.0202	1.0436	2.2714	1.1033	0.0367	1.8884	8,797	2,774	3,130
**Mean C**	5.2351	8.1649	1.2277	2.5921	1.2527	0.0314	2.1190	8,394	3,158	3,393
**Median MS**	3.6882	7.0398	1.0231	2.3685	1.0380	0.0385	1.9001	8,742	2,971	3,094
**Median C**	4.6538	8.1081	1.1885	2.6707	1.2490	0.0314	2.1024	8,403	3,137	3,331
**SD MS**	1.4863	1.6554	0.2841	0.7321	0.2936	0.0111	0.3890	0,824	0,825	0,277
**SD C**	1.9583	1.7159	0.3691	0.6051	0.2786	0.0059	0.3840	0,523	0,623	0,228
**Min. MS**	0.5605	2.4933	0.3044	0.9225	0.5396	0.0046	0.9405	7,224	1,079	2,587
**Min. C**	3.1720	4.2024	0.4670	1.0184	0.8008	0.0158	1.3817	7,440	1,559	2,888
**Max. MS**	7.1839	10.3890	1.4823	4.0717	1.9188	0.0535	2.7748	10,944	4,209	4,116
**Max. C**	10.5309	12.2885	1.9275	3.8221	1.7835	0.0447	3.4311	9,441	4,623	3,792
**T-value**	-3.0277	-2.8498	-2.3274	-2.0199	-2.1990	2.5818	-2.5090	2,493	-2,231	-4,384
***P*-value**	0.0037	0.0058	0.0233	0.0473	0.0313	0.0124	0.0145	0,015	0,029	0,001

Note: MS = MS group patients; C = control group; SD = standard deviation; Min. = minimum; Max. = maximum

Supp. Table S4: Descriptive statistics of other metabolites without counted concentrations

Metabolite	Mean MS	Mean C	Median MS	Median C	SD MS	SD C	Min. MS	Min. C	Max. MS	Max. C	T-value	*P*-value
eMethionin	8504.86	6256.73	8300.5	6154.5	2293.06	896.85	5145.5	4811	13385	8452	5.573	<0.001
Homovanilic a.	25611.18	29774.24	23860	28980	6850.19	8085.84	14365	16870	45280	48850	-2.321	0.024
Treonine	8363.92	9587.38	8235.3	9566.5	2797.24	2447,98	2823,5	3712	14440	15205	-1.965	0.053
Uridine	54333.68	73550.91	38018	57450	37916.23	46254.03	22045	25920	2E+05	2E+05	-1.897	0.063
Oxoglutaric a.	3671.48	3466.02	3723.3	3501	585.98	400.76	2146.5	2368	4895	4597	1.743	0.086
Serotonine	72399.12	36146.67	9551.3	8743	103619.34	70506.17	1712.5	2520	3E+05	3E+05	1.742	0.086
Biotine	18633.25	23614.50	16049	21000	10638.00	14333.83	6746.5	8545	55730	64085	-1.642	0.106
Palmitoleic a.	16785.92	15807.12	16475	15215	2676.63	2426.12	13145	11885	26565	23495	1.616	0.111
Dopamine	7914.79	8832.67	7763	8593.5	2566.33	2475.26	2953.2	3886	14010	13225	-1.532	0.130
Adipic a.	93639.30	98516.06	94880	100850	19305.67	10593.10	34793	75585	1E+05	1E+05	-1.342	0.185
Creatine	414855.54	444733.33	408050	410100	100129.75	98329.90	137681	3E+05	6E+05	6E+05	-1.266	0.210
Tryptophane	75840.66	71938.48	73153	71055	12870.37	13716.55	44765	50455	1E+05	1E+05	1.230	0.223
Leucine	46331.37	50641.21	43488	48665	16522.71	12977.94	18581	24640	1E+05	83070	-1.229	0.223
Adenine	208212.43	219853.18	209750	227400	44745.61	36331.27	69291	1E+05	3E+05	3E+05	-1.209	0.231
Alanine	9162.12	9821.76	9099.5	9458	2287.16	2585.61	3653.2	6084	12945	15245	-1.131	0.262
Uric a.	18416.67	16896.35	1890.66	1429.62	11654.81	8212.53	5335	4963	55645	43035	0.641	0.523
Cystine	13257.83	13426.36	13175	13210	969.36	1277.90	11430	11495	15665	17615	-0.619	0.539
Aspartate	5438.97	5558.92	5458.5	5516	918.10	726.05	2998.1	3501	7198	6947	-0.614	0.541
Palmitic a.	53262.32	54647.30	60228	55413	20906.23	8844.53	10852	29667	78285	76128	-0.372	0.712
Myristic a.	4917.08	5009.62	4617.8	4904	2478.17	1479.14	2557.5	2872	17821	8931	-0.194	0.847

Note: MS = MS patient group; C = control group; SD = standard deviation; Min. = minimum; Max. = maximum; a. = acid
